# Nanopore Hybrid Assembly of Biscogniauxia mediterranea Isolated from Quercus cerris Affected by Charcoal Disease in an Endangered Coastal Wood

**DOI:** 10.1128/MRA.00450-21

**Published:** 2021-10-21

**Authors:** Marzia Beccaccioli, Alessandro Grottoli, Luca Scarnati, Luigi Faino, Massimo Reverberi

**Affiliations:** a Department of Environmental Biology, Sapienza University of Rome, Rome, Italy; b ARSIAL, Agenzia Regionale per lo Sviluppo e l'Innovazione dell'Agricoltura del Lazio, Rome, Italy; University of Maryland School of Medicine

## Abstract

Biscogniauxia mediterranea is the causal agent of charcoal disease, affecting oak decline under the trigger of various biotic and abiotic factors, including climate change. Here, we report the genome assembly of an Italian *B. mediterranea* strain obtained using hybrid sequencing technologies combining long and short reads.

## ANNOUNCEMENT

The causal agent of charcoal disease is the fungal pathogen Biscogniauxia mediterranea. *B. mediterranea* spends part of its life cycle endophytically in twigs, bark, and leaves and less in the wood until environmental stresses induce rapid colonization of the xylem and cortex, causing the formation of cankers that accelerate the decline of oak, possibly provoking its death (1). *B. mediterranea* is widespread in Italy, as well as in other Mediterranean Basin countries and in central-western Europe (2). The Palo Laziale Wood (Latium, Italy) has suffered a serious oak decline in the last decades (1), caused by various critical factors such as the abandonment of sylvicultural practices, the rise in temperatures, the increase in edaphic salinity due to the depositions of sea winds, and the changing precipitation regimes; these factors are correlated with climate change and have triggered widespread *B. mediterranea* infection (3, 4). Here, we report the genome assembly of an Italian *B. mediterranea* strain obtained by combining hybrid data derived from long- and short-read sequencing approaches.

*B. mediterranea* strain FBL658 was isolated from stomata present on oak trunks sampled in Palo Laziale Wood. A monosporic culture was selected and plated on oatmeal agar (HiMedia). The plates were maintained in the dark at 25°C for 1 week. DNA was extracted from 30 mg of freeze-dried mycelium grown in potato dextrose broth (HiMedia) in the dark at 250 rpm for 5 days. Genomic DNA (gDNA) was extracted from *B. mediterranea* using the cetyltrimethylammonium bromide (CTAB) method (5).

A contiguous genome sequence was assembled by combining two different sequencing technologies: Oxford Nanopore Technologies (ONT) and BGI DNBseq sequencing. The ONT sequencing library was prepared according to the manufacturer’s instructions in the native barcoding genomic DNA (EXP-NBD104) protocol, while the BGI DNBseq library was prepared by BGI Europe Genomics. Approximately 400 ng of gDNA was sequenced using an ONT MinION device with no size selection, and 1 μg was sequenced by BGI Europe Genomics. The ONT MinION run produced ∼460,000 reads, base called using Guppy v3.2.4 (2.53 Gbp; coverage, ∼60×; read *N*_50_, 22.7 kbp), whereas DNBseq paired-end 2 × 150-bp (PE150) sequencing produced  ∼19 million 2 × 150-bp reads (2 × 3.00 Gbp; coverage, ∼70×). The BGI read quality check was performed using FastQC v0.11.7 (https://www.bioinformatics.babraham.ac.uk/projects/fastqc/) and trimmed using BBDuk v38.79 (https://sourceforge.net/projects/bbmap/) (quality score, ≥32; minimum length, 60). The genome sequence was assembled using Minimap2 v2.12-r849-dirty (6) and miniasm v0.3-r179 (7) and polished twice using Racon v1.3.3 (8), Nanopolish v0.11.1 (9), and Pilon v1.24 (10) for improving assembly consensus sequences with the BGI reads. Default parameters were used except where otherwise noted. The assembly quality was evaluated using QUAST v5.0.2 (11). Gene prediction was performed using MAKER v3.01.03 (12) and evaluated using BUSCO v4.1.4 (13) with the Ascomycota database (Table 1). This new assembly will be useful in comparative and evolutionary studies for planning environmental restoration actions in endangered coastal woods.

**TABLE 1:** Core metrics of the Biscogniauxia mediterranea assembly, MAKER gene prediction, and BUSCO quality analysis

*B. mediterranea* genome feature	Value
Metrics	
Total length	43,749,209
No. of contigs	18
No. of contigs (≥10,000 bp)	15
No. of contigs (≥25,000 bp)	15
No. of contigs (≥50,000 bp)	15
Largest contig (bp)	6,332,603
*N*_50_ (bp)	4,853,196
*L*_50_	5
GC content (%)	46.98
MAKER summary	
No. of predicted genes	10,444
Mean gene length (bp)	∼1,890
BUSCO^*a*^ summary (%)	
Complete BUSCOs (C)	97.4
Complete and single-copy BUSCOs (S)	97.2
Complete and duplicated BUSCOs (D)	0.2
Fragmented BUSCOs (F)	0.9
Missing BUSCOs (M)	1.7

a BUSCOs, benchmarking universal single-copy orthologs.

### Data availability.

This whole-genome assembly project has been deposited at the NCBI under BioProject accession number PRJNA727443; the Nanopore and DNBseq reads are deposited at the SRA database under accession numbers SRR14425514 and SRR14425513, respectively. This whole-genome shotgun project has been deposited at DDBJ/ENA/GenBank under the accession number JAGXTR000000000. The version described in this paper is version JAGXTR010000000.

## References

[1] Mazzaglia A, Anselmi N, Gasbarri A, Vannini A. 2001. Development of a polymerase chain reaction (PCR) assay for the specific detection of *Biscogniauxia mediterranea* living as an endophyte in oak tissues. Mycol Res 105:952–956. doi:10.1016/S0953-7562(08)61951-6.

[2] Luque J, Parladé J, Pera J. 2001. Pathogenicity of fungi isolated from *Quercus suber* in Catalonia (NE Spain). Forest Pathol 30:247–263. doi:10.1046/j.1439-0329.2000.00208.x.

[3] Barbieri M, Battistel M, Garone A. 2013. The geochemical evolution and management of a coastal wetland system: a case study of the Palo Laziale protected area. J Geochem Explor. 126:67–77. doi:10.1016/j.gexplo.2012.12.014.

[4] Thomas FM, Blank R, Hartmann G. 2003. Abiotic and biotic factors and their interactions as causes of oak decline in Central Europe. Forest Pathol 32:277–307. doi:10.1046/j.1439-0329.2002.00291.x.

[5] Punelli F, Reverberi M, Porretta D, Nogarotto S, Fabbri AA, Fanelli C, Urbanelli S. 2009. Molecular characterization and enzymatic activity of laccases in two *Pleurotus* spp. with different pathogenic behaviour. Mycol Res 113:381–387. doi:10.1016/j.mycres.2008.11.018. 19116166

[6] Li H. 2018. Minimap2: pairwise alignment for nucleotide sequences. Bioinformatics 34:3094–3100. doi:10.1093/bioinformatics/bty191. 29750242 PMC6137996

[7] Li H. 2016. Minimap and miniasm: fast mapping and de novo assembly for noisy long sequences. Bioinformatics 32:2103–2110. doi:10.1093/bioinformatics/btw152. 27153593 PMC4937194

[8] Vaser R, Sović I, Nagarajan N, Šikić M. 2017. Fast and accurate de novo genome assembly from long uncorrected reads. Genome Res 27:737–746. doi:10.1101/gr.214270.116. 28100585 PMC5411768

[9] Loman NJ, Quick J, Simpson JT. 2015. A complete bacterial genome assembled de novo using only Nanopore sequencing data. Nat Methods 12:733–735. doi:10.1038/nmeth.3444. 26076426

[10] Walker BJ, Abeel T, Shea T, Priest M, Abouelliel A, Sakthikumar S, Cuomo CA, Zeng Q, Wortman J, Young SK, Earl AM. 2014. Pilon: an integrated tool for comprehensive microbial variant detection and genome assembly improvement. PLoS One 9:e112963. doi:10.1371/journal.pone.0112963. 25409509 PMC4237348

[11] Mikheenko A, Prjibelski A, Saveliev V, Antipov D, Gurevich A. 2018. Versatile genome assembly evaluation with QUAST-LG. Bioinformatics 34:i142–i150. doi:10.1093/bioinformatics/bty266. 29949969 PMC6022658

[12] Cantarel BL, Korf I, Robb SMC, Parra G, Ross E, Moore B, Holt C, Alvarado AS, Yandell M. 2008. MAKER: an easy-to-use annotation pipeline designed for emerging model organism genomes. Genome Res 18:188–196. doi:10.1101/gr.6743907. 18025269 PMC2134774

[13] Seppey M, Manni M, Zdobnov EM. 2019. BUSCO: assessing genome assembly and annotation completeness. Methods Mol Biol 1962:227–245. doi:10.1007/978-1-4939-9173-0_14. 31020564

